# Lightweight Ultra-Wideband Absorbing Metamaterials Based on Multi-Dimensional Structural Design

**DOI:** 10.3390/ma19040803

**Published:** 2026-02-19

**Authors:** Aixiong Ge, Shaobo Qu, Baocai Xu

**Affiliations:** 1 Department of Basic Sciences, Air Force Engineering University, Xi’an 710051, China; alex202509@126.com (A.G.); qushaobo@126.com (S.Q.); 2 School of Materials Engineering, Hebei Vocational University of Industry and Technology, Shijiazhuang 050091, China

**Keywords:** structural design, microwave absorption, metamaterials, lightweighting

## Abstract

Addressing the technical bottlenecks of excessive surface density in traditional magnetic metal powder absorbers and excessive thickness in conventional foam-based absorbers, this study proposes a novel lightweight, ultra-wideband microwave-absorbing metamaterial. This metamaterial, through multi-layer and multi-dimensional structural design, has constructed a composite structure composed of a resistive film frequency-selective surface, a foam wave-absorbing medium layer and a reflective layer, achieving the controllable regulation of microwave absorption performance and the integration of structure and function. The research results show that the fabricated absorbing metamaterial achieves efficient electromagnetic wave absorption over a wide frequency band of 94 GHz under the ultra-light and ultra-thin conditions with a density as low as 0.078 g/cm^3^ and a thickness of only 4.9 mm. This study provides an effective design concept and solution for developing new lightweight, thin-layer, wide-band, and highly microwave-absorbing materials.

## 1. Introduction

Currently, the rapid evolution of fifth-generation (5G) mobile communications, the Internet of Things, and radar detection technologies has given rise to an increasingly intricate electromagnetic environment. This renders electromagnetic compatibility (EMC) and shielding critical challenges in the design of electronic devices and systems [1,2]. As a pivotal technological solution for mitigating electromagnetic interference (EMI) and improving signal integrity (SI), microwave-absorbing materials are now confronted with comprehensive performance requirements, including lightweight properties, thin-film processability, and broadband functionality [1,2,3,4].

Traditional microwave-absorbing materials—such as ferrites [5], barium titanate [6], silicon carbide [7], graphite, and conductive fibers [8]—demonstrate excellent electromagnetic absorption capabilities. Nevertheless, they commonly suffer from critical limitations: narrow absorption bandwidths, high density, and excessive thickness, which often fail to meet the demands of practical applications [9]. Moreover, while conventional magnetic metal powder absorbers deliver high absorption efficiency, their considerable areal density poses significant hurdles for deployment in lightweight scenarios [10]. In contrast, traditional foam-based absorbers, despite their low areal density, typically require substantial thickness to achieve desired performance metrics and thus fall short of the rigorous lightweight and broadband absorption requirements of emerging technologies [11].

Metamaterials have emerged as a burgeoning research hotspot in the field of microwave-absorbing materials, owing to their unique advantages in regulating electromagnetic properties [12]. Their distinctive design paradigm—characterized by tunable absorption bands—facilitates structural integration with conventional materials. This innovative approach not only broadens the absorption bandwidth of absorbers and enhances absorption efficiency but also achieves a significant reduction in material weight and thickness. Nevertheless, a majority of current metamaterial absorbers still suffer from the limitation of narrow absorption bands, which restricts their further application in broadband scenarios [13].

Furthermore, to break through the performance bottleneck brought by a single material or a single structure, multi-dimensional structural design is regarded as an effective way to broaden the microwave absorption bandwidth [14]. This approach entails constructing material or structural units with distinct electromagnetic response characteristics in three-dimensional space; by harnessing the synergistic loss mechanisms among these units, efficient electromagnetic absorption is achieved across multiple frequency bands concurrently, thereby stitching or merging these discrete bands into a continuous broadband spectrum. For instance, Qu N et al. [15] realized ultra-wideband absorption spanning 2–40 GHz in a 9.3 mm-thick MOF/Fe metamaterial via a composite design based on two-dimensional material grain boundaries. Similarly, Zhang T et al. [16] developed a multi-scale engineered Ag/N-rGO composite aerogel that achieved an effective absorption bandwidth (EAB) of 7.04 GHz (covering the Ku-band), which was further extended to 14.64 GHz through periodic structural engineering. Nevertheless, a multitude of existing metamaterial absorbers still grapple with narrow absorption bands or overly intricate structural configurations, restricting their full-band applicability and impeding the simultaneous optimization of low density, thin thickness, and ultra-wide bandwidth [17,18].

This study proposes a lightweight ultra-wideband absorptive metamaterial based on a multi-dimensional structural design. By integrating the lightweight characteristics of foam-type microwave-absorbing material with the spatial structural regulation capabilities of metamaterials, the proposed design constructs a gradient composite structure consisting of a “resistive film frequency-selective surface–foam absorptive medium layer–metallic reflective layer.” This approach effectively addresses the limitations of conventional absorbers regarding frequency bandwidth, thickness, and areal density, thereby providing valuable insights for the development of novel lightweight, high-performance ultra-wideband absorbers.

## 2. Preparation and Characterization

### 2.1. Material Simulation

The CST MICROWAVE STUDIO was adopted as the material simulation software. In this study, the radar frequency band was set from 1 to 110 GHz. The boundary condition settings were as follows: periodic boundary conditions (unit cell) were configured in the X and Y directions to simulate an infinite periodic array structure; the Z direction was set as an open boundary (open add space). The frequency-domain finite integration solver was selected. Finally, the field monitor was configured to record properties such as the electromagnetic field distribution, and the solver parameters were set to ensure the accuracy and efficiency of the calculation.

### 2.2. Material Preparation

Preparation of Zeolitic Imidazolate Framework-67 (ZIF-67) and Cobalt-based Layered Double Hydroxide (Co_x_-LDH) composite materials (ZIF-67/Co_*x*_-LDH): All four ZIF-67/Co_*x*_-LDH (x = Fe, Mn, Zn, Ni) materials were synthesized using the same process: using ZIF-67 as the cobalt source, and reacting with Fe, Mn, Zn, and Ni salts at two molar ratios (1:4 and 1:1) to generate CoFe-LDH, CoMn-LDH, CoZn-LDH, and CoNi-LDH precursors. Taking ZIF-67/CoNi-LDH as an example, 200 mg of the pre-synthesized ZIF-67 (0.688 mmol) was dispersed in 20 mL of ethanol solution containing 0.688 mmol of hexahydrate nitrate nickel [Ni(NO_3_)_2_·6H_2_O], and reacted under 300 rpm magnetic stirring for 30 min. After centrifugation to collect the product, it was washed with ethanol three times, and the final ZIF-67/CoNi-LDH precursor obtained was vacuum-dried at 70 °C for 12 h.

The preparation of absorbing paste: Mix ZIF-67/Co_*x*_-LDH composite materials, flame retardants, polyurethane water-based adhesive, mold inhibitors, light stabilizers, thickening agents, dispersants, curing agents, antioxidants, and water in a certain proportion, evenly mix, and control the viscosity at 1000~1200 mPa•s. The resulting absorbing paste is prepared.

Preparation of foamed polyurethane: Based on polyurethane foaming materials, the selective immersion method of absorbing agent paste is used to fabricate absorbing conical layers, with and without paste immersion, on a single substrate. Then, select a perforated polyurethane foaming material with a certain pore size, and cut it into the required thickness and area for future use.

Preparation of resistive film: Based on the technology of preparing and regulating the performance of conductive carbon paste films, first, a resist film substrate with the required pattern was fabricated through laser etching. Then, using the YS3050Y semi-automatic screen printing machine, the conductive carbon paste patterns with the predetermined resistance values were precisely printed onto the foam medium substrate, thereby completing the preparation of the resist film layer. The printing accuracy of the screen was 0.2 mm, which met the preparation requirements for such resist film metamaterial samples.

Finally, a high-conductivity metal film was adhered to the bottom of the foamed polyurethane, and a complete metamaterial was ultimately obtained.

### 2.3. Material Characterization and Testing

The microstructure of the metasurface was observed using an MLRA-LMS field emission scanning electron microscope (FE-SEM, TESCAN, Brno, Czech Republic) to verify the structural integrity of the prepared patterns. The density was determined by measuring the mass and volume via the Archimedes’ principle, with the reported value representing the average of three independent measurements. The reflectivity of the metamaterial samples in the frequency range of 1–120 GHz was measured using the arch method reflectivity test. The measurement method is as follows: The radar reflectivity of the metamaterial sample is tested using the dual-antenna arc-shaped reflection method. The test system mainly consists of a pair of parallel standard broadband horn antennas, which serve as the transmitting antenna and the receiving antenna, respectively, and are set up at a certain height above the sample. Due to the extremely wide frequency band, the test was conducted in a segmented manner, and corresponding standard gain horn antennas were selected based on different frequency bands: for the 1–18 GHz frequency band, a broadband double-ridge horn antenna (model: LB-10180) was used; for the 18–26.5 GHz, 26.5–40 GHz, 40–60 GHz, 60–75 GHz, and 75–90 GHz frequency bands, the corresponding standard gain horn antennas (models: LB-1826, LB-2640, LB-4060, LB-6590, LB-7590, respectively) were used; for the 90–120 GHz frequency band, a WR-08 standard gain horn antenna (model: LB-90120) was used. The test system was equipped with the corresponding millimeter-wave spread spectrum module to generate high-frequency signals. The system controls the signal transmission, reception, data acquisition, and processing through the Anritsu MS4644A vector network analyzer (Anritsu, Morgan Hill, USA) and its accompanying test software. This system has an adjustable incident angle function. By changing the relative position and angle between the transmitting antenna and the receiving antenna, the incident angle of the electromagnetic wave relative to the sample surface can be flexibly set, thereby achieving the measurement of the sample under different incident conditions of 200 mm × 200 mm to meet the basic requirements of far-field testing. To ensure the reliability and repeatability of the measurement data, a calibration piece is used to fully double-port calibrate the vector network analyzer before each formal test to eliminate system errors. During the test, a flat high-conductivity metal plate and an “air reference” without a sample are used as blank controls. Through data processing, background reflection and a system inherent response can be effectively deducted, thereby improving the accuracy of the reflectivity test results.

## 3. Results and Discussion

### 3.1. Structural Design

**Figure 1:**
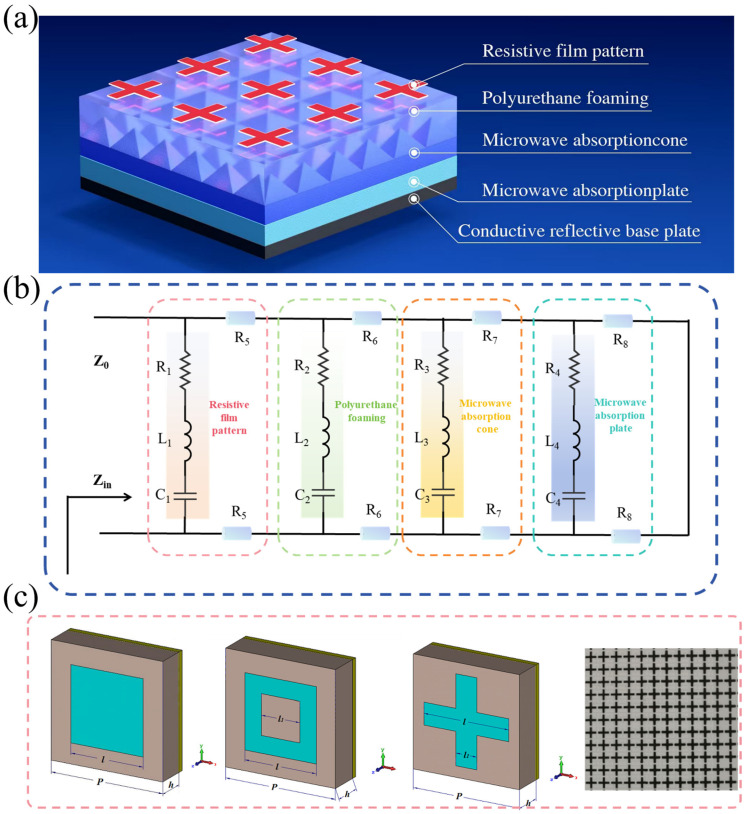
Schematic diagram of multi-dimensional structure wideband absorbing material structure. (**a**) Overall structural schematic diagram; (**b**) Equivalent circuit diagram of the absorber; and (**c**) Schematic diagram of the superstructure structural unit.

The core innovation of this solution resides in the integration of the lightweight properties of foam-type microwave-absorbing material and the structural design flexibility of metamaterials. Through multi-layer and multi-dimensional structural optimization, a functional composite with gradient-progressive electromagnetic characteristics is constructed. Serving as a multi-band, wide-spectrum stealth absorber, this multi-dimensional broadband absorptive material comprises five distinct layers with tailored properties and functions, as depicted in Figure 1a. The outermost layer (Layer 1) is a metamaterial pattern layer, fabricated by laser cutting carbon fiber films with specific electromagnetic parameters. Variations in pattern geometry, resistance, and dimensional parameters endow the metamaterial structure with corresponding electromagnetic loss characteristics, laying the foundation for frequency-selective absorption. The second layer is a plain polyurethane foam, which provides air impedance matching to facilitate the penetration of most incident electromagnetic waves into the material interior, minimizing reflection at the air-material interface.

The third layer consists of pyramidal polyurethane foam absorbers. The polyurethane foam matrix is impregnated with an absorber agent featuring high electromagnetic wave absorption efficiency; the arrayed pyramidal structure enables impedance grading and progressive electromagnetic wave attenuation, further enhancing the absorption performance through geometrically induced energy dissipation (the absorbing agent is composed of ZIF-67/Co_*x*_-LDH composite material, flame retardant, polyurethane water-based adhesive, antifungal agent, light stabilizer, thickener, dispersant, curing agent, antioxidant and deionized water). The fourth layer is a flat polyurethane foam absorber impregnated with an electromagnetic wave absorber that exhibits high attenuation capability for low-frequency electromagnetic waves. This layer provides additional attenuation for the internally propagating electromagnetic waves and can be configured into a multi-layer composite structure with gradient transitions, depending on the specific requirements for thickness and weight. The fifth layer (bottom layer) is a conductive substrate acting as an electromagnetic wave reflection layer, ensuring that the unabsorbed internal electromagnetic waves are reflected back into the upper absorption layers for secondary dissipation. Collectively, the surface patterned array layer, intermediate foam material layers, and bottom reflection layer form a metamaterial absorber capable of broadband electromagnetic wave absorption. This multi-dimensional, multi-mechanism design strategy ingeniously synergizes the lightweight advantage of foam materials with the electromagnetic control capabilities of metamaterials. The gradient distribution of electromagnetic parameters across the functional layers creates a complete energy loss pathway of “impedance matching → progressive absorption → total reflection,” enabling the absorber to achieve high-efficiency broadband electromagnetic wave absorption while maintaining lightweight and ultra-thin characteristics. The equivalent circuit of this metamaterial absorber is shown in Figure 1b and Figure S1. The coupling design between layers was determined based on preliminary systematic simulations. The corresponding unit simulation model and the simulation results of key parameters for different layers are shown in Figures S2 and S3, respectively. Building upon this validation of feasibility, the study further optimized the structural design.

Firstly, the resistive film pattern layer was designed and optimized systematically. In the design process, three simple pattern units—square, cross, and square ring—were selected as candidate configurations for the resistive film metamaterial unit. Schematic diagrams of the three designed resistive film metamaterial structures are presented in Figure 1c. The key structural parameters of the resistive film metamaterial include the unit period (P), pattern unit dimensions (l and l_1_), and dielectric layer thickness (h). The reflectivity of the three metamaterial configurations was calculated using the frequency-domain solver in CST electromagnetic simulation software, and the structural parameters of each resistive film unit were optimized iteratively. After the optimization process, the optimal structural parameters for the three patterned metamaterials were determined. Simulation results indicated that all three designed unit metamaterials achieved dual-peak broadband absorption effects following parameter optimization. Among the candidates, the square-cell metamaterial exhibited the highest absorption peak but suffered from a relatively narrow bandwidth. In contrast, the square-ring and cross-cell metamaterials demonstrated broader absorption bands, with the cross-cell configuration performing most prominently—it featured the widest absorption band and a high relative bandwidth, whose absorption range covers multiple frequency bands. Consequently, the cross-cell pattern was ultimately selected as the optimized design for the resistive film layer.

**Figure 2:**
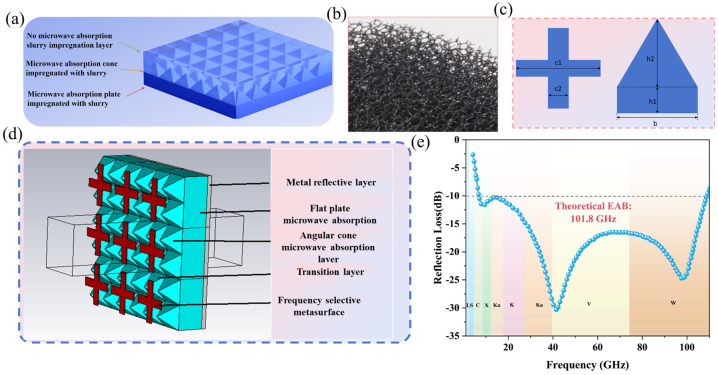
Unit Structure and Simulation Results. (**a**) Schematic of the foamed polyurethane layer, (**b**) Microscopic image of the absorbing foam, (**c**) Schematic of structural dimensions, (**d**) Simulation image, and (**e**) Simulation results.

Secondly, systematic design and optimization were conducted for the intermediate lightweight polyurethane foam layer. A polyurethane filter sponge with high open-cell porosity and flexible bendability was selected as the substrate material, offering excellent physical properties and structural adaptability. Building upon this foundation, a selective impregnation process using absorber slurry was employed to construct separate absorber cone layers, impregnated layers, and non-impregnated layers on the same substrate, achieving integrated structure–function integration (Figure 2a). The key to this process lies in the selection of the fillers in the absorbing agent. To balance lightness and high absorption efficiency, we abandoned the magnetic metal absorbing agents with excessive density, and overcame the problem that the purely dielectric materials have limited absorbing capacity. Instead, we used the self-made ZIF-67/Co_*x*_-LDH composite material as the electromagnetic dual-loss type absorbing filler, which combines dielectric loss and magnetic loss mechanisms and significantly enhances the electromagnetic wave attenuation ability. The microstructure of the overall absorbing foam is shown in Figure 2b. In the modeling analysis, to simplify computational complexity, the angularly conical foam structure with non-uniform electromagnetic parameter distribution in practice was approximated as an isotropic homogeneous medium. Equivalent electromagnetic parameters were obtained using the transmission reflection method combined with an air coaxial line fixture and waveguide test system. The simulation optimization was conducted with the criteria of achieving the optimal bandwidth for reflection loss ≤ −10 dB and ensuring that the overall thickness does not exceed 5 mm. Through optimized simulation of unit cell dimensions, the total thickness of the absorptive functional layer was determined to be 4.9 mm, the metal reflective layer thickness to be 0.5 mm, the planar absorptive layer thickness set to h_1_, the pyramid height h_2_ = 4.9 − h_1_, with the pyramids shaped as regular tetrahedrons. with base edge length b. The surface frequency-selective pattern features a cross-shaped resistive film structure with edge length c_1_ and line width c_2_ (Figure 2c). Following full-band optimization simulations (Figure 2d), the optimal structural parameters were finalized as follows: planar layer thickness h_1_ = 2.7 mm, pyramidal height h_2_ = 2.2 mm, base edge length b = 2.1 mm, cross dimensions c_1_ = 14.5 mm and c_2_ = 2.4 mm, with the resistive film’s sheet resistance set at 18.26 Ω/□. This metamaterial exhibits excellent absorbing performance over a wide frequency range, as shown in Figure 2d. The theoretical effective absorbing bandwidth of this metamaterial can reach 101.8 GHz, covering the C, X, Ku, K, Ka, V, and W frequency bands. It achieves the coordinated design goal of ultra-wideband efficient absorption and lightweight structure.

### 3.2. Experimental Verification

**Figure 3:**
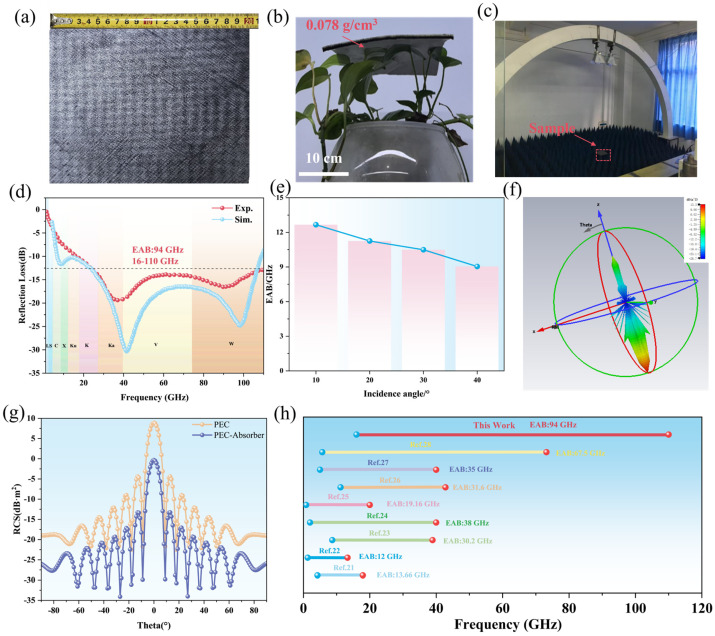
Experimental tests of metamaterial samples. (**a**) Sample appearance, (**b**) Lightweight demonstration, (**c**) Testing device, (**d**) Measured results, (**e**) Absorption bandwidth under different incident angles, (**f**,**g**) RCS attenuation performance simulation, and (**h**) Performance comparison with other works [21,22,23,24,25,26,27,28].

After determining the structural parameters, the designed metamaterial absorbing body was processed and fabricated. The appearance of the fabricated sample is shown in Figure 3a. Its density is only 0.078 g/cm^3^, demonstrating an extremely low-density characteristic. It can be easily placed on the surface of the blade without causing significant deformation, as shown in Figure 3b, indicating excellent lightweight properties. Furthermore, this metamaterial exhibits outstanding comprehensive mechanical properties. Its compressive load-bearing capacity can exceed 5000 times its own weight, and its tensile load-bearing capacity is over 2000 times its own weight. It also possesses good flexibility and structural toughness, as shown in Figure S4. After the preparation of the multi-layer foam metamaterial absorbing body sample, systematic tests were conducted on its electromagnetic wave absorption performance in a microwave dark room. The test environment was surrounded by a wave-absorbing cone to reduce interference. The reflectance measurement system used the arch method reflectivity test, which included an Agilent vector network analyzer, transmitting and receiving horn antennas, and a computer-controlled and data acquisition software package. The overall configuration is shown in Figure 3c. During the test, the sample was placed on the test platform, and its reflectance data was obtained in real time through the software. The measured results are shown in Figure 3d. The comparative analysis of the simulation results shows that the trend of the simulated reflection loss curve is in good agreement with the measured reflection loss curve, and the error of the absorption peak is relatively small. Although there are certain deviations in the intensity and position of the resonance peaks between the two. However, this is a common phenomenon in many existing studies on metamaterial stealth, where there are certain deviations between the simulation and experimental results in the high-frequency range [19,20]. The reasons are mostly related to the idealization of material parameters, the simplification of boundary conditions, the dispersion characteristics, and the inherent errors of the environment. Although there are local differences, the measured and simulated curves are in good agreement in the overall trend. This metamaterial absorber achieves efficient electromagnetic wave absorption over a wide frequency band of 94 GHz under the condition of ultra-light and ultra-thin thickness of only 4.9 mm. The effective absorption frequency band is 16–110 GHz, covering five bands including Ku, K, Ka, V, and W. It realizes low-thickness and low-density ultra-wideband microwave absorption. Further examination of the absorption characteristics under different incident angles is shown in Figure 3e. Under the incident angles of 10°~40°, the absorber maintains an effective absorption frequency band of no less than 80 GHz, demonstrating good angle insensitivity. The radar cross section (RCS) of this metamaterial was simulated at 60 GHz, as shown in Figure 3f,g. As can be seen from the figure, even at the 60 GHz frequency point where the reflection loss is relatively low, this metamaterial still exhibits excellent RCS attenuation characteristics. The metamaterial absorber prepared in this work was compared with various types of current metamaterial in terms of the effective absorption frequency band, as shown in Figure 3h and Table S1. The metamaterial prepared in this work has an ultra-wide effective absorption frequency band, which is much higher than that of all the reported types of absorptive metamaterials.

### 3.3. Analysis of Microwave Absorption Mechanisms

**Figure 4:**
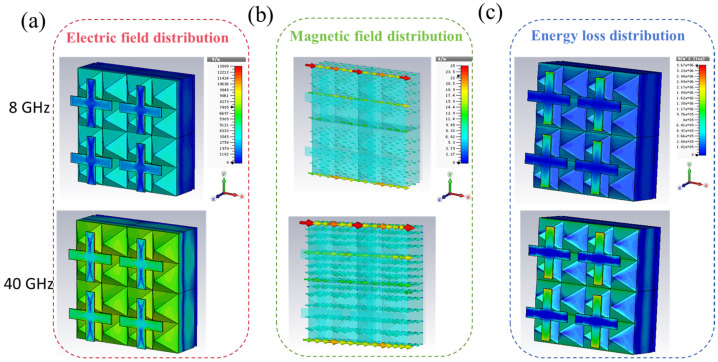
Electromagnetic field distribution and energy dissipation distribution of the metamaterial absorber. (**a**) Electric field distribution, (**b**) Magnetic field distribution, and (**c**) Energy dissipation distribution.

To further clarify its microwave absorption mechanism, the working principle of this multi-dimensional structure absorber is analyzed as follows: This structure consists of a resistive membrane frequency-selective surface on the surface layer, a foam-absorbing medium in the middle layer, and a metal reflective layer at the bottom. Its broadband absorption property is attributed to the collaborative effect of the loss mechanisms in each layer and the gradient matching of the overall impedance. The preliminary equivalent circuit simulation indicates (Figures S1, S2, S3, and S5), this design can effectively regulate the surface impedance of the structure by adjusting the equivalent parameters of the surface resistive membrane units—the equivalent capacitance is related to the surface electric field distribution, and the equivalent inductance is related to the surface current distribution—so as to achieve a good match with the free-space impedance over a wide frequency range, thereby significantly reducing reflection and increasing the absorption bandwidth. To more intuitively reveal the loss mechanism and field distribution characteristics of this composite metamaterial at key frequency points, we selected the frequencies corresponding to two typical absorption peaks in the absorption spectrum (f = 8 GHz and f = 2 GHz), and analyzed the electromagnetic field distribution and energy loss density under the TE wave mode. The results are shown in Figure 4. Figure 4a reveals that the electric field primarily concentrates around the resistive film patterns. At the low-frequency absorption peak, the electric field is more localized, mainly distributed along the upper and lower edges of the cross-shaped patterns. At the high-frequency absorption peak, the electric field spreads around the edges of both the resistive film and the absorptive corner cone of the dielectric layer. Figure 4b reveals that the magnetic field distribution is opposite to the electric field. The magnetic field concentrates at the center of the resistive film pattern and within the absorptive foam medium. At the low-frequency absorption peak, the magnetic field strength is very high in the central region of the resistive film, decreasing with distance from the pattern. As frequency increases, the magnetic field strength at the center of the resistive film decreases, while the magnetic field strength within the dielectric layer—particularly in the absorptive foam medium—significantly increases. Figure 4c reveals that the energy loss distribution shows concentrated losses at the low-frequency absorption peak within the absorptive foam’s absorptive cone edge region and the absorptive foam medium layer. At the high-frequency absorption peak, losses within the absorptive foam medium increase. The energy loss distribution on the resistive film closely follows the electric field distribution, indicating that the loss in the resistive film is primarily ohmic loss. In contrast, the loss in the absorptive foam medium results from the combined effects of electric and magnetic losses. Calculations yielded the energy loss curves for each structure within the composite metamaterial. The energy loss of the incident electromagnetic wave is primarily distributed between the resistive film and the absorptive foam medium. As frequency increases, the energy loss in the resistive film first increases and then decreases, while the loss in the absorptive foam medium first increases, then decreases, and finally gradually increases again. The incorporated absorptive foam layer enhances energy dissipation across both low- and high-frequency bands, compensating for the limitations of resistive film loss and further improving the absorptive performance of resistive film-based metamaterials. The composite metamaterial based on a dual-layer structure of absorptive pyramids and absorptive plates represents a simple and effective layered composite approach. By incorporating absorptive foam electromagnetic media into the resistive film-based metamaterial structure, it introduces additional types of magnetic loss on top of the resistive film’s ohmic loss. This enhances loss capabilities across both low- and high-frequency bands, further expanding the absorptive bandwidth of resistive film-based metamaterials.

## 4. Conclusions

This work combines the design concept of metamaterials with foaming-type absorbing materials to construct a multi-layer and multi-dimensional composite structure consisting of a frequency-selective surface of resistive film, a foam-absorbing medium layer, and a reflective layer. The research results show that this absorbing metamaterial can achieve efficient electromagnetic wave absorption in a wide frequency band ranging from 16 GHz to 110 GHz under the condition of extremely low surface density of 0.078 g/cm^3^ and a thickness of only 4.9 mm. The effective absorbing bandwidth can reach 94 GHz, and it also exhibits good angle insensitivity. This work not only effectively solves the problem of excessive density or thickness of traditional absorbing materials, but also significantly improves the absorbing performance and integrates the structural function, providing important practical guidance for the design and application of new microwave absorbing materials. Future work will focus on addressing practical challenges such as the environmental stability (e.g., thermal resistance and moisture absorption) of the foam layer and the scalability of the “selective impregnation technique”, whose complexity may increase large-scale production costs compared to simpler foam absorbers.

## Data Availability

The original contributions presented in this study are included in the article/Supplementary Material. Further inquiries can be directed to the corresponding author.

## Supplementary Materials

The following supporting information can be downloaded at: https://www.mdpi.com/article/10.3390/ma19040803/s1, Figure S1: Equivalent circuit diagram in the simulation software; Figure S2: Schematic diagram of multi-layer metamaterial absorbing structure unit; Figure S3: Simulation results of different layers. (a) First layer, (b) Second layer, (c) Third layer, (d) Fourth layer; Figure S4: Display of the mechanical properties of metamaterials. (a) Flexibility, (b) Compressive resistance, (c) Tensile resistance; Figure S5: The equivalent impedance of each layer in the multilayer metamaterial. (a) Real part of impedance matching, (b) Impedance matching imaginary part; Table S1: The performance comparison of the samples prepared in this work with those of other previously reported works.
