# Supplementary material for: Lightweight Ultra-Wideband Absorbing Metamaterials Based on Multi-Dimensional Structural Design
Source: Materials (Basel). 2026 Feb 19;19(4):803. doi: 10.3390/ma19040803 (PMC12941481; doi:10.3390/ma19040803)
Supplement: Supplementary file 1 [file materials-19-00803-s001.zip › materials-4139825-supplementary.pdf]

Supplementary Information

Lightweight Ultra-Wideband Absorbing Metamaterials Based on Multidimensional Structural Design

Aixiong Ge[1], Shaobo Qu[1], Baocai Xu[2]✉

1. Department of Basic Sciences, Air Force Engineering University, Xi'an, 710051, China

2. Hebei Vocational University of Industry and Technology, Shijiazhuang, 050091, P. R. China

✉Corresponding author: xbcabcd@163.com (Baocai Xu)

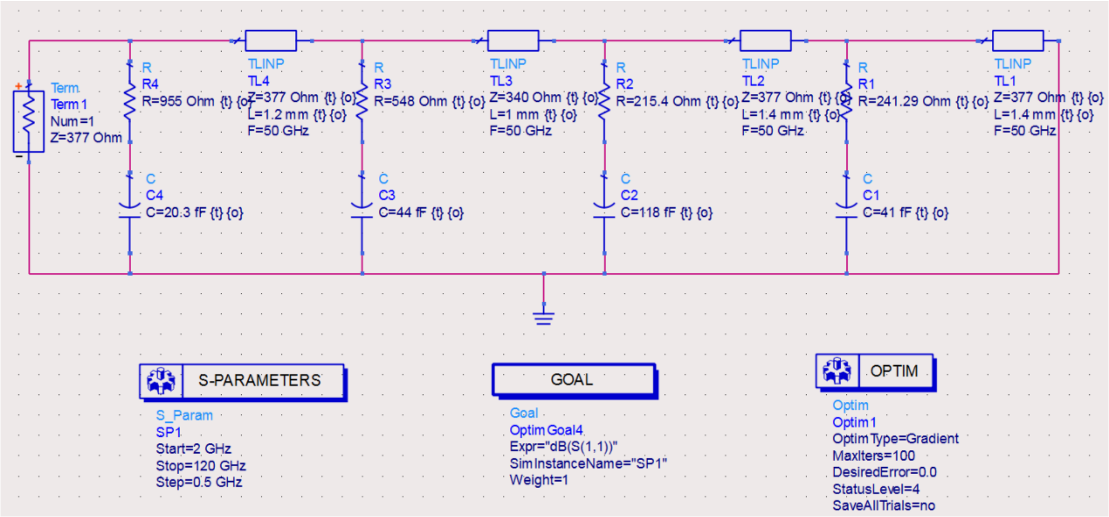

Figure S1. Equivalent circuit diagram in the simulation software

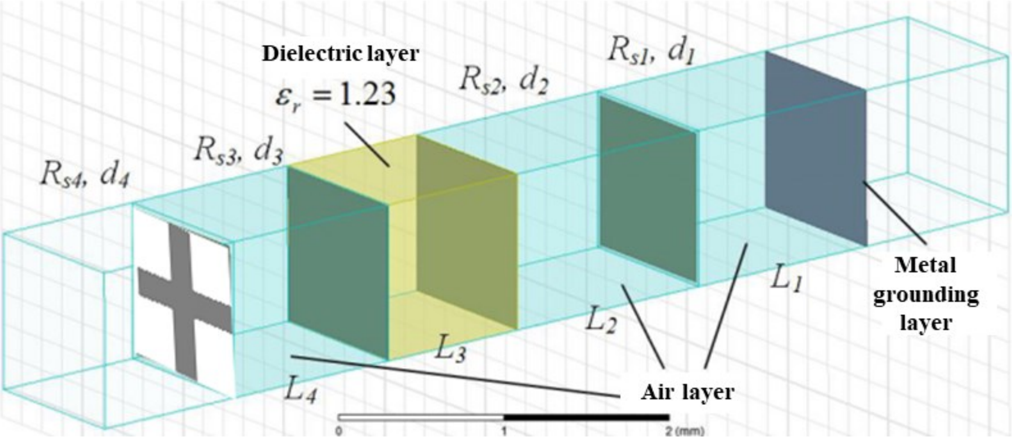

Figure S2. Schematic diagram of multi-layer metamaterial absorbing structure unit

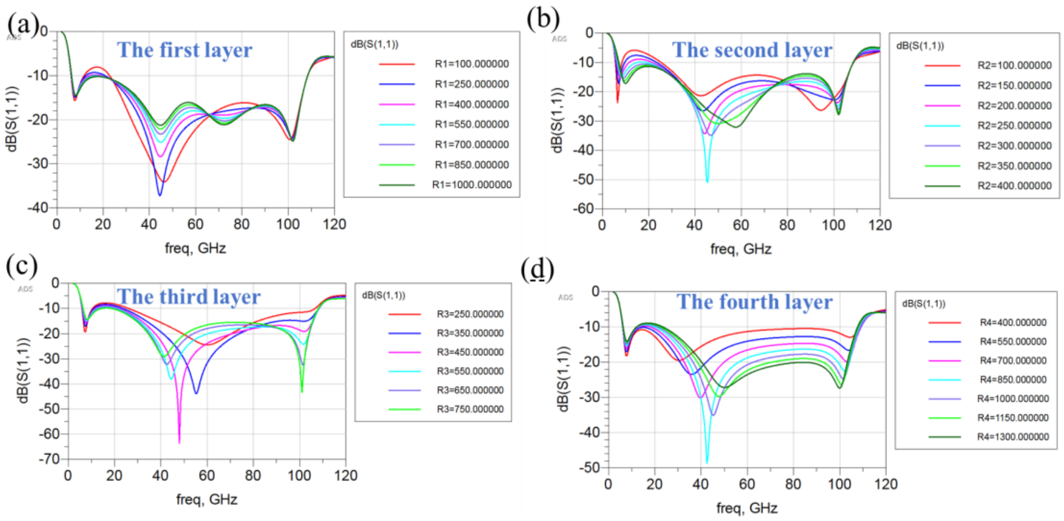

Figure S3. Simulation results of different layers

(a) First layer, (b) Second layer, (c) Third layer, (d) Fourth layer

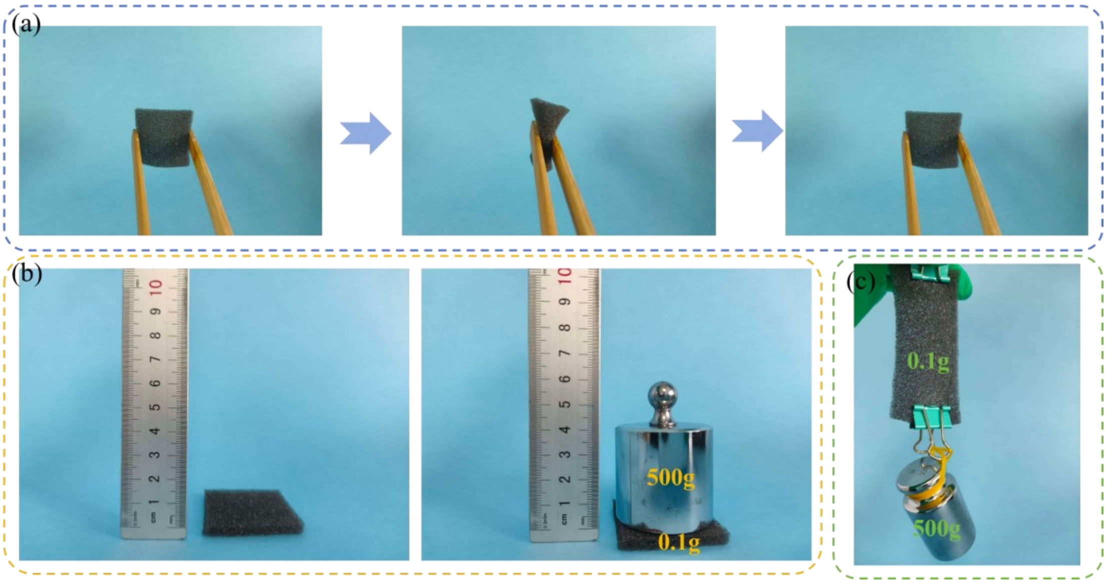

Figure S4. Display of the mechanical properties of metamaterials

(a) Flexibility, (b) Compressive resistance, (c) Tensile resistance

(a)

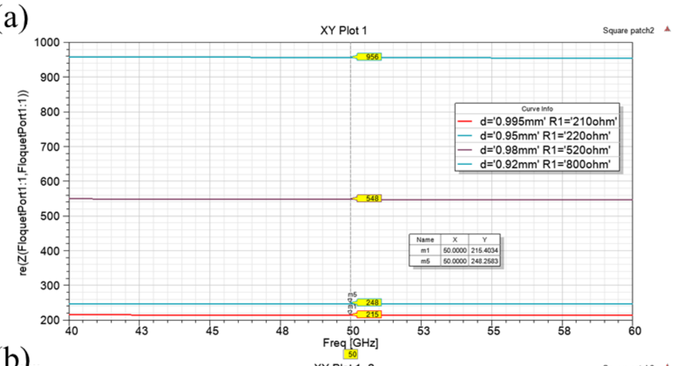

(b)

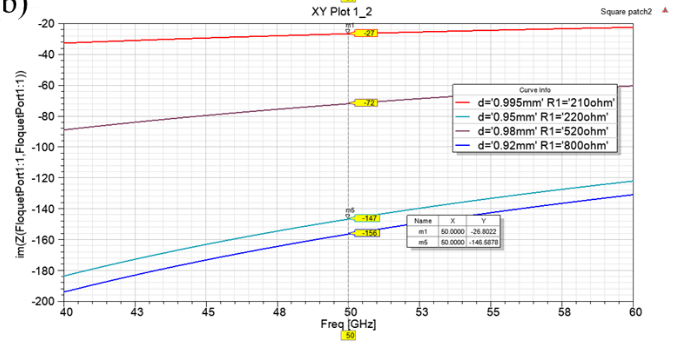

Figure S5. The equivalent impedance of each layer in the multilayer metamaterial (a) Real part of impedance matching, (b) Impedance matching imaginary part

Table S1. The performance comparison of the samples prepared in this work with those of other previously reported works

**Sample name**	**Thickness/ mm**	**Effective absorption bandwidth/GHz**	**Density/ g/cm^3**	**Mechanical properties**	**References**
GAF MMA	5.77	13.66	0.085	Flexibility	[1]
Sample	30.52	12	0.067	\	[2]
Sample	4.2	30.2	\	Flexibility	[3]
Sample	5	38	\	\	[4]
Sample	26.5	19.16	\	\	[5]
PNIPAM gel	\	35	\	Flexible, Pressure-resistant, Tensile-resistant	[6]
Sample	5.9	67.5	\	\	[7]
**Sample**	**4.9**	**94**	**0.078**	**Flexible, Pressure-resistant, Tensile-resistant**	**This work**

References

[1] Zhou Y, Zhou B, Jin S, et al. Highly reliable and ultra-wideband metamaterial absorber based on graphene-assembled films for extremes[J]. Carbon, 2024, 229: 119534.

[2] Dong F Y, Niu C, Zhang M, et al. A lightweight ultra-wideband metasurface microwave absorber[J]. Advanced Materials Technologies, 2025, 10(7): 2401493.

[3] Dai H, Li S, Dong P, et al. Design of an ultra-wideband transparent wave absorber[J]. Materials,

2023, 16(17): 5962.

[4] Wang G, Li D, Liao W, et al. Agile Stealth: Bioinspired Metamaterials with Continuous Dynamic Tuning[J]. Advanced Materials, 2025: e11070.

[5] Hou Z, Cheng J, Kong X, et al. Design of Ultra-Broadband Metamaterial Absorber with Angle-insensitive Characteristics Covering the L, S, C, X, and Ku Bands[J]. IEEE Antennas and Wireless Propagation Letters, 2025.

[6] Hou Z, Cheng J, Kong X, et al. Design of Ultra-Broadband Metamaterial Absorber with Angle-insensitive Characteristics Covering the L, S, C, X, and Ku Bands[J]. IEEE Antennas and Wireless Propagation Letters, 2025.

[7] Yang J, Guo Y, Li X, et al. Fabry–Pérot Matching Lossy Metasurface for Coordinated, Adaptive, and Ultra-Broadband Visible-Infrared-Radar Compatible Camouflage[J]. Advanced Materials, 2025: e17422.
